# Should “Matrescence” be included in Psychiatric manuals? – A review

**DOI:** 10.1192/j.eurpsy.2026.11945

**Published:** 2026-07-31

**Authors:** A. Lourenço, M. Ribeiro, A. Duarte, J. Romão, J. Revez Lopes

**Affiliations:** ULSSM, Lisboa, Portugal

## Abstract

**Introduction:**

he birth of a child is fundamental to the continuity of humanity, and therefore, it is essential to prioritize the care of pregnant women. While our knowledge and technologies are advancing rapidly, the investment and protection of maternity are progressing at a slower pace. In recent decades, fields like sociology, anthropology, and psychiatry have become more attuned to the profound changes inherent to motherhood. As a result, the concept of “matrescence” has emerged to describe the many transformations that occur in a woman’s life during this period.

**Objectives:**

To review the literature about “matrescence”.

**Methods:**

We performed a MEDLINE search using the keyword: matrescence. We only included studies with full text published in English.

**Results:**

In our Medline review, we found a total of 22 full articles using the keyword “matrescence” to date. Although only 15 articles had been published between 2006 and the end of 2024, a notable surge was observed in 2025, with 7 new articles published in this year alone, of which only five were reviews in the total sample. Matrescence can be compared to adolescence, as both represent significant and irreversible transitions—marked by profound transformations and the discovery of a new sense of self. Women experience physical, emotional, psychological, and social changes during this period. While physical changes are easily noticeable, psychological shifts are more subtle and harder to identify, as they involve a transformation of identity often accompanied by doubts, anxiety, and guilt. This experience is deeply shaped by cultural beliefs and social realities, and is recognized as a major biosocial life event. Studies also show that women undergo structural brain changes during this time, not only due to hormonal fluctuations but also because of actual alterations in brain architecture, which biologically prepare them for new maternal responsibilities. This transition involves an increased cognitive load during the peripartum period, requiring continuous adaptation; however, research suggests that when these demands are sustained across the lifespan, they may result in increased cognitive reserve in later life. Despite this biological adaptation, motherhood is fraught with challenges and pressures—not only from the mother herself but also from her family and society. It is a period of heightened risk for mental health issues, as studies have shown, with significant implications for the mother, child, and family.

**Conclusions:**

Although professionals are increasingly aware of the changes associated with motherhood, this understanding is not yet widespread, and some psychiatric manuals still fail to reflect it. While the recent growth in publications suggests an interest that is rapidly increasing in the concept, matrescence remains a relatively understudied area overall, highlighting the need for further studies and greater awareness of this transformative phase.

**Disclosure of Interest:**

None Declared

